# Chikungunya virus and armed forces: relevance and vaccine prospects

**DOI:** 10.1007/s10096-024-05028-x

**Published:** 2025-01-06

**Authors:** Jonas Schmidt-Chanasit, Ralf Matthias Hagen, Hagen Frickmann

**Affiliations:** 1 Department of Arbovirology and Entomology, Bernhard Nocht Institute for Tropical Medicine, 20359 Hamburg, Germany; 2 Faculty of Mathematics, Informatics and Natural Sciences, Universität Hamburg, 22609 Hamburg, Germany; 3 Department of Microbiology and Hospital Hygiene, Bundeswehr Central Hospital Koblenz, 56070 Koblenz, Germany; 4 Institute of Medical Microbiology, Immunology and Parasitology (IMMIP), University Hospital Bonn, 53127 Bonn, Germany; 5 Department of Microbiology and Hospital Hygiene, Bundeswehr Hospital Hamburg, 20359 Hamburg, Germany; 6 Institute for Microbiology, Virology and Hygiene, University Medicine Rostock, 18057 Rostock, Germany

**Keywords:** Chikungunya virus, Military deployment, Force health protection, Prevention, Vaccination

Stahlman and Langton [1] reported chikungunya virus (CHIKV) infections in U.S. armed forces members between 2016 and 2022. There were 8 cases over this period, with 2 cases per year except for 2020 and 2021, when strict COVID-19 measures were in place. Only one case was deployment-related, acquired in 2019 in Djibouti during a local outbreak. Notably, one individual required hospitalization, and another experienced prolonged sequelae for over 12 months. Stahlman and Langton’s findings align with our previous review [2], indicating that CHIKV infections during military deployments in endemic areas are rare. However, during local outbreaks, such as the one in Djibouti [1], the risk for soldiers matches that of the local population [2]. Given the prolonged disease courses in infected soldiers [1, 2], leading to repatriation and lost workdays, preventive measures are necessary for deployments in chikungunya outbreak areas.

Briefly summarized, chikungunya is a mosquito-borne viral disease caused by CHIKV, a single-stranded positive-sense ribonucleic acid (RNA) virus belonging to the genus *Alphavirus* [3]. Following initial discovery in Tanzania in 1952, outbreaks have now been reported across all continents except Antarctica. In Africa, CHIKV is maintained in the environment as part of a sylvatic cycle between monkeys, and multiple Aedes mosquitoes [3]. In Asia, the Americas and more recently in Europe, the virus is transmitted in an urban cycle between *Aedes aegypti* and *Ae. albopictus* mosquitoes and humans [3]. During outbreaks, human beings serve as the primary amplification hosts for the virus [3].

More than 70% of CHIKV infection in humans will result in chikungunya. The acute phase of chikungunya is characterized by the presence of a high fever, a rash, a headache and severe joint pain, which often results in the affected individual being incapacitated for a period of days to weeks [3]. This poses a significant health risk to affected populations, including military personnel deployed in endemic regions [4]. Nearly 50% of acute chikungunya patients will enter the chronic phase. The chronic symptoms associated with chikungunya, such as persistent arthralgia and fatigue, can persist for months to years, which has the potential to significantly impair operational readiness and the overall effectiveness of military units [4]. Treatment of the acute phase is limited to painkillers and nonsteroidal anti-inflammatory drugs [3]. Physical activities tend to aggravate the joint inflammatory process, contributing to local damage, thus prolonging the clinical condition [3]. Human CHIKV infection can be diagnosed using molecular and serological methods. Virus isolation in cell culture or detection of the virus genome by RT-PCR can be used successfully in the first week of illness [3]. The detection of anti-CHIKV IgM and IgG is possible with sufficient reliability from the second week of illness [3].

As a result of climate change, the “Asian tiger mosquito” *Ae. albopictus* is expected to spread further, increasing the risk of future chikungunya outbreaks worldwide. The short time span between the start and end of localized outbreaks is a major obstacle to effective outbreak management. Therefore, rapid response mechanisms, such as point-of-care diagnostics, local control of vectors, but also the use of vaccines, are crucial. Valneva's chikungunya vaccine IXCHIQ, which was authorized by the European medicines agency (EMA) in July 2024, could help control outbreaks [5]. IXCHIQ is a live attenuated vaccine that induces immunization after a single intramuscular administration. The vaccine has been modified by reverse genetics to insert a deletion of 60 amino acids in the nsP3 (non-structural) protein. This modification leads to attenuation of the vaccine without compromising its immunogenic properties. IXCHIQ has undergone Phase 1 and Phase 3 clinical trials, demonstrating the safety and immunogenicity of the vaccine. The final conservative endpoint agreed was serological response rate (SRR), defined as CHIKV neutralizing antibody titers ≥ 150 determined by micro-plaque reduction neutralization testing (µPRNT50) [5]. In the pivotal study, IXCHIQ demonstrated robust immunogenicity with an SRR of 98.9% on day 29, which significantly exceeded the threshold of 70% set by the FDA [5]. Six months after vaccination, the SRR remained high at 96.3%, with no differences observed between age groups. In participants under 65 years of age, the SRR was 96.7%, while it reached 94.8% in participants over 65 years of age [5]. These findings suggest that the vaccine can provide effective protection against chikungunya. The safety profile of IXCHIQ was also evaluated as part of the Phase 3 trial, which showed that most adverse events were mild or moderate. Serious adverse events occurred in only 2% of subjects who received the vaccine compared to 0.1% in the placebo group [5]. Herpes zoster and Guillain-Barré syndrome were not observed in the vaccine group and thus, the overall tolerability of the vaccine was good and no life-threatening adverse events related to the vaccine were reported. The results suggest that IXCHIQ has an acceptable safety profile for widespread use.

It is imperative that military forces at increased risk of CHIKV infections be vaccinated against CHIKV for the following reasons: The acute and chronic symptoms of CHIKV have the potential to significantly disrupt military operations [4]. As so far best described for 662 French military policemen during the Reunion Island outbreak, chronic pain in joints and bone with considerable and partly long-lasting impact on active duty were reported by the vast majority of infected service members with only a minority of asymptomatic infections [2]. Therefore, ensuring that soldiers at increased risk are vaccinated can prevent such disruptions and maintain operational readiness. Military personnel are often deployed to areas with ongoing CHIKV transmission, which increases the risk of infection [4]. Thereby, benefit-harm-balancing of mission-adapted vaccine prevention should generally consider the epidemiological likeliness of infection events and the fact that promising therapeutic options for infection-associated arthritis like treatment with hydroxychloroquine and methotrexate are available. In case of mission-specific balancing results favoring vaccine prophylaxis, vaccination provides a protective barrier against the disease, reducing the incidence of chikungunya among troops. Furthermore, the global mobility of armed forces increases the risk of introducing CHIKV to non-endemic areas, which could potentially lead to local outbreaks.

## Data Availability

No datasets were generated or analysed during the current study.
